# Interpretable‐AI‐Based Model Structural Transfer Learning to Accelerate Bioprocess Model Construction

**DOI:** 10.1002/bit.70026

**Published:** 2025-07-18

**Authors:** Alexander W. Rogers, Fernando Vega‐Ramon, Amanda Lane, Philip Martin, Dongda Zhang

**Affiliations:** ^1^ Department of Chemical Engineering The University of Manchester Manchester UK; ^2^ Unilever R&D Port Sunlight Wirral Liverpool UK

**Keywords:** bioprocess kinetics, digital twin, interpretable machine learning, knowledge discovery, model‐based design of experiments

## Abstract

Determining accurate kinetic models for new biochemical systems is time‐intensive, requiring experimental data collection, model construction, validation, and discrimination. Traditional black‐box machine learning‐based transfer learning methods leverage prior knowledge but lack interpretability and physical insights. To address this, we propose a novel model structural transfer learning approach that combines symbolic regression with artificial neural network feature attribution. The method enables automatic structural modification of an inaccurate or low‐fidelity mechanistic model developed for one system when being applied to another system. Through a comprehensive in silico case study, our framework successfully adapted a kinetic model from one biochemical system to a different but related one, improving predictive accuracy. Moreover, the framework can significantly accelerate model identification when being integrated with model‐based design of experiments. By comparing the old and new model structures, physical insight can be obtained, altogether highlighting the framework's potential for advancing automated knowledge discovery and facilitating high‐fidelity predictive digital twin design for novel biochemical processes.

## Graphical abstract

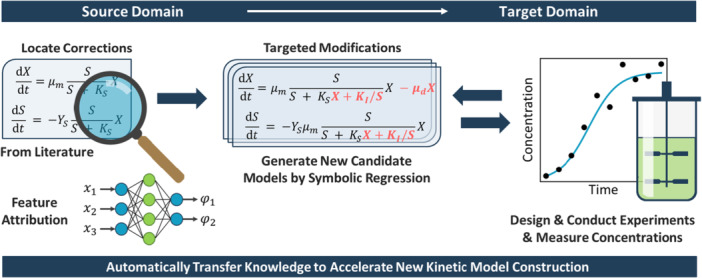

## 1 Introduction

Developing accurate models to predict bioprocess dynamics is critical for commercial process design, scale‐up, and control (G. Wang et al. 2020). Kinetic models, typically described by ordinary differential equations (ODEs), are constructed from prior knowledge of reaction mechanisms and kinetics, with each parameter and term possessing physical significance. However, constructing accurate kinetic models is often challenging due to incomplete or uncertain knowledge of the underlying biochemical reaction networks (Almquist et al. 2014). Human practitioners will typically explore different model structures adapted from literature or personal experience of similar systems, but it can still take weeks to identify an accurate model structure (da Paz et al. 2025; Villadsen et al. 2011). In contrast, data‐driven techniques, such as machine learning methods (e.g., artificial neural networks [ANNs], Gaussian processes, and random forests) (Duong‐Trung et al. 2023; Helleckes et al. 2023), are relatively quick to deploy and excel at capturing complex bioprocess dynamics without direct knowledge of the governing physical laws, but lack interpretability and require substantial, high‐quality datasets that are time‐consuming to generate (Fish`er et al. 2020; Tsopanoglou and Jiménez Del Val 2021). Currently, there are two highly successful techniques for addressing the limitations of mechanistic and data‐driven models, respectively: hybrid modeling and transfer learning.

Hybrid models typically formulate mass balances with simplified reaction terms to capture the main relationships, while embedded data‐driven terms describe the unknown kinetics as lumped time‐varying parameters (Agharafeie et al. 2023; Von Stosch et al. 2014). This accounts for the reality that many constants in traditional kinetic models are intrinsically transient, unknown functions of the dynamic state variables (e.g., biomass, substrate, and product concentration). For strongly hysteretic bioprocesses, these unknown functions can also take as input the previous state and control trajectory (Lu et al. 2025; Smiatek et al. 2021). However, to avoid overfitting, only the smallest number of data‐driven terms can be placed within the kinetic model structure. Their placement will induce bias in the functions discovered and impact predictive capability (Hastie et al. 2017; Rogers et al. 2023). Second, these data‐driven functions remain opaque, undermining trust among practitioners who require deeper mechanistic understanding, and create bottlenecks where regulatory authorities increasingly require explainability and traceability (Di Bonito et al. 2024).

Transfer learning addresses the limitations of data‐driven approaches by updating a pretrained model from a related system to accelerate new model development. Pre‐training on a related bioprocess and fine‐tuning with minimal data from the new bioprocess can markedly improve dynamic predictions, helping to speed up screening across mutant strains (Rogers et al. 2022) and digital twin deployment across bioreactor configurations (Riezzo et al. 2025). Likewise, transfer learning, combined with techniques like subdomain alignment (B. Wang et al. 2024) and meta‐learning (Helleckes et al. 2024), can reduce prediction error and experimental burden when working with microbial and mammalian cell cultures. However, current transfer learning approaches only update the kinetic or data‐driven parameters, leaving the kinetic model structure unchanged. Whereas oftentimes structural changes are necessary, and reflect key differences in the underlying metabolism, providing physical insights into how the source and target bioprocesses differ (Choudhury et al. 2022; Mahanty 2023). This is crucial, since transfer learning is only effective when the source and target systems share sufficient similarity (Ehrig et al. 2024; Tahir et al. 2024), where greater interpretability could provide an early indication of whether transfer learning will succeed when the model is used for prediction.

In recent years, the push for interpretable, parsimonious models has driven the development of digital methods for discovering governing equations automatically (Schmidt and Lipson 2009; Udrescu et al. 2020). The sparse identification of nonlinear dynamics (SINDy) algorithm (Brunton et al. 2016) applies sparse linear regression to identify ODEs from a predefined library of candidate functions, an approach that has since been extended to partial differential equations (Rudy et al. 2017) and to implicit, rational functions under noise (Kaheman et al. 2020; Mangan et al. 2016). Similarly, sparse identification techniques have been used to elucidate macroscopic stoichiometry and bioprocess kinetics (Pimentel et al. 2024). However, sparse identification techniques depend on crafting a suitably expressive library of basis functions. A more flexible approach is to represent equations as unidirectional graphs (i.e., expression trees) built from primitive mathematical operators (e.g., addition, subtraction, multiplication, division, or logarithmic functions). Genetic programming by tournament selection begins with a randomly generated population of expressions, then iteratively promotes the best‐fitting expressions to the next round, applying mutations whereby new branches are added, pruned, or swapped in the expression trees (Cranmer 2023). Although SR has shown promise in discovering chemical (Servia et al. 2023) and biochemical (Forster et al. 2024) kinetic equations, without prior knowledge, SR can produce non‐interpretable local approximations. While there exist other approaches to SR (Biggio et al. 2021; Kamienny et al. 2022), SR's fundamental lack of any optimality guarantees (Virgolin and Pissis 2022), makes it challenging to discover from scratch, accurate yet parsimonious expressions for complex systems with many possible input variables.

Inspired by how human practitioners typically explore model structures adapted from literature or personal experience with similar systems, we propose a novel framework for automatically adapting the structure of an existing kinetic model from related systems to rapidly generate accurate and interpretable models for new bioprocesses. This method will use the source equations as a mechanistic backbone to fit a series of hybrid correction models, systematically locating where the minimum number of ANN correction terms need embedding. Then interpretable approximations of these ANN terms will be built by SR and substituted to represent structural corrections, and if necessary, time‐varying kinetics by replacing constants with functions of the state variables. Where new terms follow structures familiar to those in the bioprocess kinetic modeling literature, these will be interpreted accordingly, enabling comparison between the source and new bioprocess kinetics. This approach should suit small‐data applications, such as dynamic bioprocess modelling where only a small number of experiments (i.e., six–eight) are available (Ündey et al. 2010). This approach should also reduce the reliance on high‐frequency measurements of the dynamic state variables (i.e., two times per day rather than every 2 h), which is another limitation of current methods (Forster et al. 2024; Pimentel et al. 2024; Servia et al. 2023), while being quicker computationally than if ODE solvers were embedded with the SR fitness function (Narayanan et al. 2022).

Section 2 will detail the methodology, then Section 3 will outline the case study used to demonstrate the framework. Next, Section 4 will present the results and discussion for two scenarios: one in Section 4.1 where prior knowledge is abundant but partially incorrect, and another in Section 4.2 where it is correct but incomplete, representing a common practical trade‐off when considering how much inductive bias should be carried over from other systems and literature. In addition, model structural transfer learning will be integrated with model‐based design of experiments (MbDoE) to reduce the number of experiments required to discover the correct kinetic equations for a new bioprocess. Section 4.3 will then finally provide some practical guidance on gauging source‐target similarity, structural identifiability, and hyperparameter selection.

## 2 Methodology

A kinetic model represents the rate of change, dy/dt, of (bio)chemical species concentrations, y, with respect to time, t, as a system of ODEs, f(y,θ), with parameters θ. Our framework aims to adapt the structure of a source model, $f_{s}\left(y,\theta_{s}\right)$, from a related system to yield a transfer model, $f_{t}\left(y,\theta_{t}\right)$, that more accurately fits and make predictions on a new target system.

**Figure 1:**
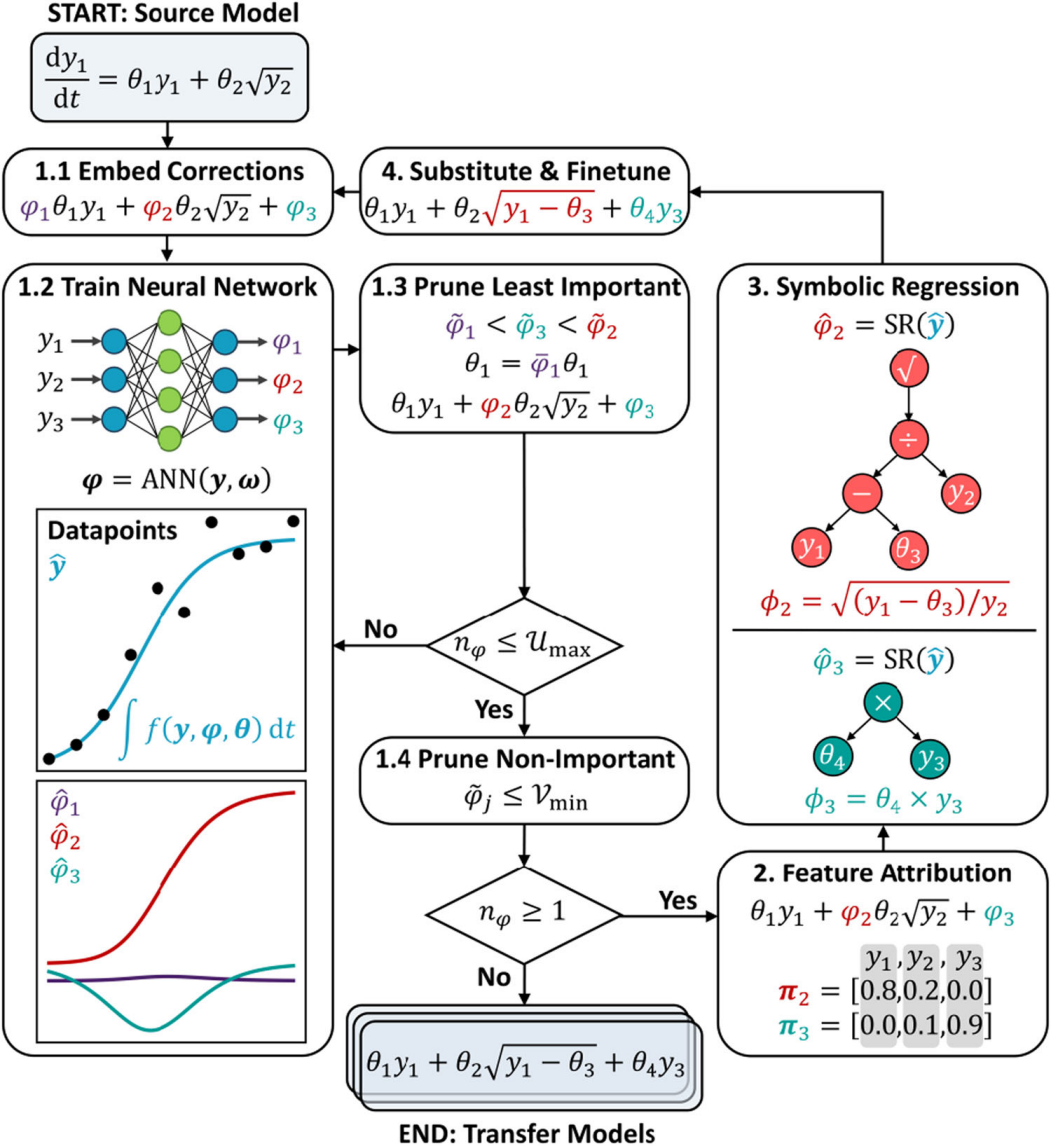
Artificial neural network (ANN) attribution guided symbolic regression (SR) for dynamic systems. The method takes equations for the source domain and data for the target domain, and outputs candidate transfer models for model‐based design of experiments.

As illustrated in Figure 1, the framework comprises four steps. In Step 1, ANN correction terms are embedded into the original mechanistic model (Step 1.1), and then these are trained to map the state variables, y, to the corrections, φ (Step 1.2). Iteratively, the least important corrections are pruned based on their variance, $\tilde{\varphi }$, in (Steps 1.3 and 1.4). In Step 2, feature attribution identifies the most influential input features for each remaining correction term, yielding weighting vectors $\pi =\left[\pi_{1},\pi_{2},\pi_{3}\right]$ for the state variables $y=\left[y_{1},y_{2},y_{3}\right]$. In Step 3, SR constructs the symbolic corrections, ϕ, using these feature weightings. Finally, in Step 4 the symbolic corrections are substituted back into the source model, $f_{s}\left(y,\theta_{s}^{\prime }\right)$, for final parameter fine‐tuning, producing the transfer model $f_{t}\left(y,\theta_{t}\right)$. In Figure 1 and throughout this article, $\hat{y}$ will denote the fitted states, while $\hat{\varphi }$ are the fitted correction terms, and ϕ are symbolic approximations of these correction terms generated by SR.

### 2.1 ANN‐Based Algorithm for Locating Structural Corrections

The goal of Step 1 is to pinpoint where corrections are most needed and to discover their functional form. We will use an example to illustrate: consider the ground‐truth target model, $g_{t}\left(y,\theta_{t}\right)$, parameterized by $\theta_{t}$, in Equation (1a) and the source model, $f_{s}\left(y,\theta_{s}\right)$, in Equation (1b), parameterized by $\theta_{s}$, where $\theta_{t}\neq \theta_{s}$. By inserting $\phi_{1}\left(y\right)=\left[1/(y_{1}+\theta_{3})\right]$ and $\phi_{2}\left(y\right)=\left[y_{1}/y_{4}\right]$ into Equation (1b) and re‐optimizing the parameters, Equation (1c) can match Equation (1a). These are the *simplest* symbolic corrections, which we can deduce here by inspection.

(1a) $$g_{t}\left(y,\theta_{t}\right)=\theta_{1}\frac{1}{y_{2}+\theta_{2}}\frac{1}{y_{1}+\theta_{3}}-\theta_{4}\frac{y_{3}}{y_{4}}$$

(1b) $$f_{s}\left(y,\theta_{s}\right)=\theta_{1}^{\prime }\frac{1}{y_{2}+\theta_{2}^{\prime }}-\theta_{4}^{\prime }\frac{y_{3}}{y_{1}}$$

(1c) $$f_{t}\left(y,\theta_{t}\right)=\theta_{1}\frac{1}{y_{2}+\theta_{2}}\left[\frac{1}{y_{1}+\theta_{3}}\right]-\theta_{4}\frac{y_{3}}{y_{1}}\left[\frac{y_{1}}{y_{4}}\right]$$

(1d) $$f_{t}\left(y,\theta_{t},\omega \right)=\theta_{1}\frac{1}{y_{2}+\theta_{2}}\varphi_{1}\left(y,\omega \right)-\theta_{4}\frac{y_{3}}{y_{1}}\varphi_{2}\left(y,\omega \right)$$

However, because in reality the structure of the corrections are unknown a priori, we begin by inserting an ANN, φ(y,ω), in their place. This single multi‐input multi‐output ANN maps the input features, y, to the chosen correction terms, φ. If necessary, the ANN can take any additional relevent control inputs such as temperature, pH or light intensity. The resulting model, $f_{t}\left(y,\theta_{t},\omega \right)$, is a hybrid model, combining mechanistic equations with data‐driven corrections. The ANN, parameterized by a set of weights and biases, ω, is optimized simultaneously with the mechanistic model parameters, θ, to fit measurements of the dynamic state variables, y, (i.e., biomass, substrate and product concentrations over time for batch bioprocesses) using the one‐step hybrid model training approach (Pinto et al. 2022).

(2a) $$\min_{\theta ,\omega }\;\frac{1}{n_{i}n_{e}}\sum_{i=1}^{n_{i}}\sum_{e=1}^{n_{e}}{\left\|{\hat{y}}_{i,e}\left(\theta ,\omega \right)-y_{i,e}\right\|}^{2}+n_{\varphi }P\left(y,\omega \right)$$

s.t.

(2b) $$\hat{\varphi }\left(\hat{y},\omega \right)=\text{ANN}\left(\hat{y},\omega \right)$$

(2d) $${\hat{y}}_{i,e}\left(\theta ,\omega \right)=y_{0}^{e}+\int_{0}^{t_{i}}f_{t}\left(y,\theta_{t},\omega \right)dt$$

(2e) $$P\left(y,\omega \right)=\lambda_{1}{\left\|\frac{\partial \hat{\varphi }}{\partial \hat{y}}\right\|}^{2}+\lambda_{2}{\left\|\hat{\varphi }\left(y,\omega \right)-b\right\|}^{2}$$

The system of ODEs, $f_{t}\left(y,\theta_{t},\omega \right)$, were numerically integrated in Equation (2d) from the initial batch conditions, $y_{0}^{e}$, at time t=0 until the end‐of‐batch time, $t=t_{i}$. Then, the mean‐square error between the measured states, $y_{i,e}$, and predicted states, ${\hat{y}}_{i,e}$, was computed in Equation (2a), averaged over each measurement time interval, i, and experiment, e, in the training dataset. During numerical integration, automatic differentiation tracked the gradients in the objective with respect to θ and ω, enabling Adaptive Moment Estimation to iteratively update the parameters and minimize the mean‐square error objective. A five‐hidden‐neuron ANN was trained with an exponential learning rate scheduler until convergence, a weight decay of 0.001 and gradient clipping to prevent exploding gradients. This was implemented in the *Python* package *PyTorch* 2.2.2 (Paszke et al. 2017), with all other hyperparameters at their defaults.

**Figure 2:**
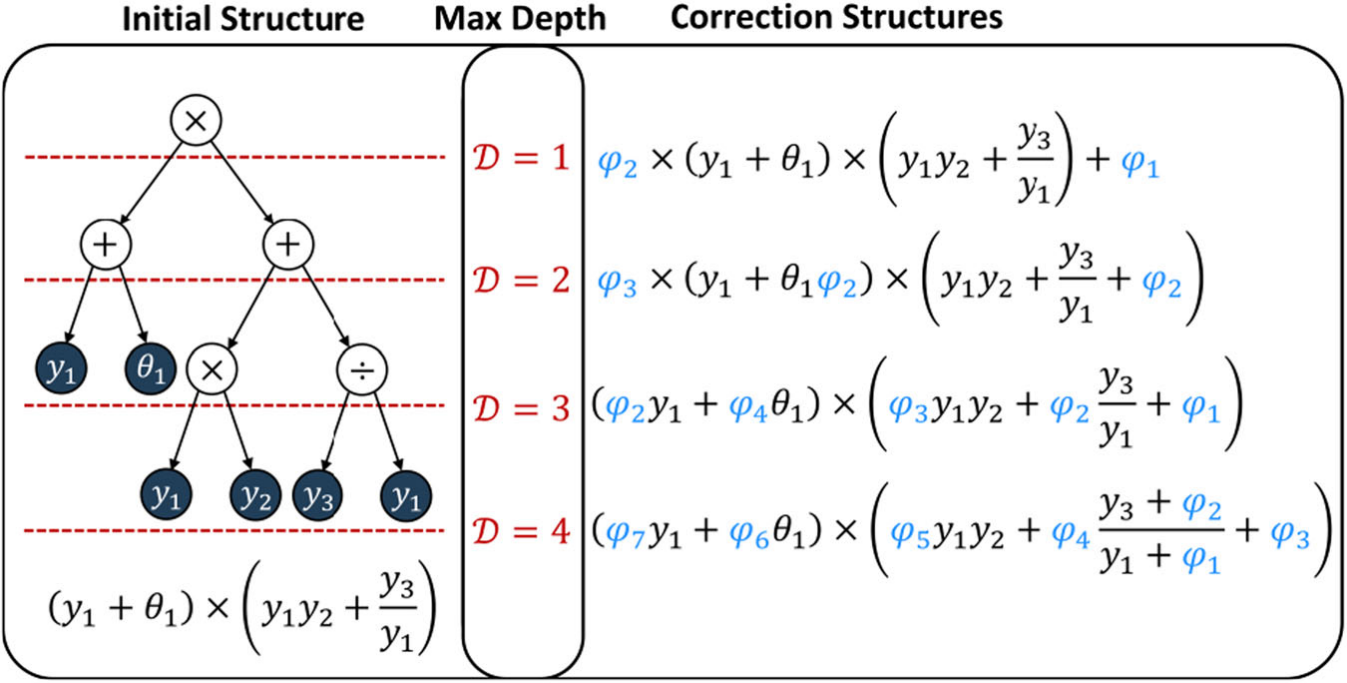
Illustration of how correction terms are embedded for different maximum depths $D_{\max}$. Shown left is an example initial equation structure, while on the right are the resulting equations with the correction terms, $\varphi_{j}$, embedded.

To begin with, it is not known where the corrections should be located. In principle, they could be placed anywhere, but this increases the risk that the corrections discovered will be complex and nonintuitive. Therefore, while they were initially placed throughout the mechanistic equations, representing possible correction locations, they must then be iteratively pruned. To systematize their placement, correction terms were placed at the leaves of the equations' truncated expression tree representations. Figure 2 illustrates how $D_{\max}$ sets the truncation depth. In this study, $D_{\max}=3$, based on the complexity of the source equations.

To mitigate overfitting a penalty, P(y,ω), weighted by the number of correction terms, $n_{\varphi }$, was appended to the objective function in Equation (2a). Defined in Equation (2e), P(y,ω) was composed of two components. The first component was the sum of the squares of the elements in the Jacobian, $\partial \hat{\varphi }/\partial \hat{y}$, aiming to limit variation in the fitted correction terms, $\hat{\varphi }$, with respect to the fitted state variables, $\hat{y}$, ensuring only truly influential input variables are retained. This also helps promote the discovery of simpler corrections that are functions of only a few input features. The second component pushes additive corrections toward zero and multiplicative ones toward unity, helping to isolate genuinely necessary correction locations. Each element in the binary vector, b, corresponds to a different correction term.

Subject to this penalty, unimportant corrections will likely have smaller variance in their outputs over the range they are evaluated during batch bioprocess simulation. The mean and variance of a correction term, ${\tilde{\varphi }}_{j}$, is calculated in Equation (3) by evaluating the correction function, $\varphi_{j}\left({\hat{y}}_{i,e},\omega \right)$, at each time index, i, for each experiment, e, in the training data set. Through an iterative procedure of re‐fitting the hybrid correction model in Equation (2) and recomputation of the variances, the least important corrections are removed, until their number, $n_{\varphi }$, fell below a pre‐set threshold. In this study, that threshold was set to $U_{\max}=3$, based on the number of dynamic state variables. Any corrections with a variance ${\tilde{\varphi }}_{j}{<\;V}_{\min}=0.1$ were also removed. This all takes place using equations adapted for normalized time and state variables. The hyperparameters $\lambda_{1}$, $\lambda_{2}$, and $V_{\min}$ were calibrated against the set of 36 independent expressions in Section S6. On an NVIDIA RTX A1000 GPU, hybrid model fitting takes ~20 s, so 10 pruning iterations, for example, would take only a few minutes. Custom symbolic manipulations using *SymPy* (Meurer et al. 2017) automated expression manipulations.

(3a) $${\bar{\varphi }}_{j}=\frac{1}{n_{i}n_{e}}\sum_{i=1}^{n_{i}}\sum_{e=1}^{n_{e}}\varphi_{j}\left({\hat{y}}_{i,e},\omega \right)$$

(3b) $${\tilde{\varphi }}_{j}=\frac{1}{n_{i}n_{e}}\sum_{i=1}^{n_{i}}\sum_{e=1}^{n_{e}}{\left(\varphi_{j}\left({\hat{y}}_{i,e},\omega \right)-{\bar{\varphi }}_{j}\right)}^{2}$$

### 2.2 Attribution Guided Symbolic Regression (SR) to Construct Correction Equations

Once Step 1 has located and fitted the hybrid correction models, SR was used to build symbolic approximations for each of the ANN corrections. A new data set for SR was generated for each correction term, j, by evaluating the ANN corrections, $\varphi_{j}\left({\hat{y}}_{i,e},\omega \right)$, at each time index, i, for each experiment, e, in the training data set. SR then constructs expressions, $\phi_{j}\left(y,\theta \right)$, correlating ${\hat{y}}_{i,e}$ with $\varphi_{j}\left({\hat{y}}_{i,e},\omega \right)$ by minimizing the mean square error objective function in Equation (4). Tournament selection promoted and mutated candidates from a population of expressions using the *Julia* package *SymbolicRegression* 1.01 (Cranmer 2023). Over 1000 generations, and ~5 min on a 12th Generation Intel i7‐12700H CPU, a population of 1200 expressions converged. A more detailed introduction to SR is provided in Section S4.

During SR the maximum complexity, $C_{\max}$, defined as the total number of operators, variables, and constants, is constrained. The impact of $C_{\max}$ will be discussed further in Section 4.3.3 but for the case studies in this research $C_{\max}=7$. In addition, Integrated Gradients (Sundararajan et al. 2017) was used to quantify the importance of each input feature to the ANN corrections. These feature attributions were then used to uniquely weight the probability of adding or removing certain features during SR, helping to reduce the search space of possible expressions for complex systems with many possible input features (Rogers et al. 2025). For details on the calculation of the feature weightings, see Section S3.

(4) $$\min_{\phi ,\theta }\frac{1}{n_{i}n_{e}}\sum_{i=1}^{n_{i}}\sum_{e=1}^{n_{e}}{\left(\phi \left({\hat{y}}_{i,e},\theta \right)-{\hat{\varphi }}_{i,e}\right)}^{2}$$

At the end of SR, the fittest individuals at each complexity level, $C_{k}$, are arranged onto a Pareto front such that $C_{k}<C_{k+1}$. Each candidate expression, $\phi_{k}$, indexed by k, is given a score, $S_{k}$, using the negated derivative in their log‐loss, $\log L_{k}^{\text{SR}}$, with respect to complexity, $C_{k}$ (Cranmer 2023). Expressions with a higher score, $S_{k}$, will represent elbow points on the Pareto front, representing candidates that balance well fitting accuracy and complexity. The selected expressions are finally substituted back into the source equations, yielding the updated target model, then the parameters, θ, are fine‐tuned to compensate for any mismatch between the approximate symbolic corrections. During parameter finetuning, there exists an option to drop any redundant terms in the expression by promoting sparsity by L2 regularization of the parameters, followed by removal of small parameters. For details on the definition of the final parameter finetuning optimization problem, see Section S5.

### 2.3 Integrating SR and MbDoE for Model Discrimination

There can be different model structures that fit the observed data similarly well; when there is noise in the measurements, the correct model structure can become indistinguishable from incorrect model structures. MbDoE for model structure discrimination maximizes information gain from newly designed experiments by selecting conditions that maximize the difference in the predicted responses among the model candidates (Franceschini and Macchietto 2008). The general SR‐MbDoE framework (Rogers et al. 2024) was adapted to leverage model structure transfer learning.

In Section 2.2, SR generated a Pareto set of possible expressions for each correction; we shall denote each of these expressions using $\phi_{j,k}$, where j and k index the correction term and position in the Pareto set, respectively. For each correction term, j, the top two scoring expressions were selected, with the choice of how many expressions to select being determined empirically. Combinations of these are then substituted into the equations, such that if there were three correction terms, each with two possible symbolic candidates, for example, then $n_{m}=2^{3}$ overall model candidates, $f_{t}^{m}\left(y,\theta_{t}^{m}\right)$, would be constructed.

New experiments, parameterized by $z^{*}$, are designed by maximizing the objective, J(z), in Equation (5a). The process trajectory is simulated using each model, m, in Equation (5b), retrieving the state variable vector, ${\hat{y}}_{i}^{m}$, at each measurement time point, i. In Equation (5c), the generalized Hunter–Reiner criterion (Hunter and Reiner 1965), used as the objective for model discrimination, maximizes the difference in the predicted responses. Later, the design variable, z, will be the initial biomass and substrate concentration (i.e., $y_{0}=z$) but can be any set of design variables, while model uncertainty with respect to z was assumed constant (i.e., $\sigma^{2}\left(z\right)=1$).

(5a) $$z^{*}=\underset{z}{\text{argmax}}J\left(z\right)$$

s.t.

(5b) $${\hat{y}}_{i}^{m}=y_{i-1}+\int_{0}^{t_{i}}f_{t}^{m}\left(y,\theta_{t}^{m}\right)dt$$

(5c) $$J\left(z\right)=\sum_{i=0}^{n_{i}}\sum_{m=1}^{n_{m}-1}\sum_{n=m+1}^{n_{m}}\frac{{\left\|{\hat{y}}_{i}^{m}-{\hat{y}}_{i}^{n}\right\|}^{2}}{\sigma^{2}\left(z\right)}$$

**Figure 3:**
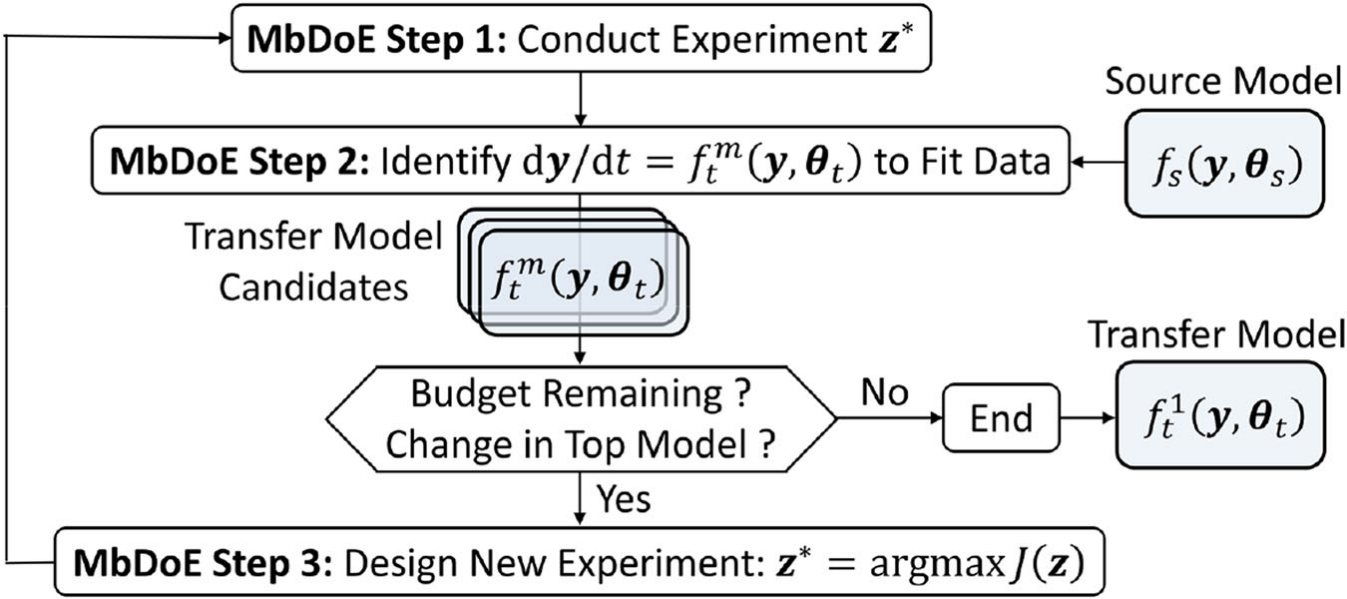
General SR‐MbDoE flowchart for proposing kinetic model expressions.

All together in Figure 3: Step 1 conducts the designed experiments; Step 2 builds a set of different candidate transfer models, drawing on the source model; then, Step 3 designs the next experiment by optimizing an objective function. MbDoE iterates until the experimental budget is exhausted or the equations from one iteration to the next cease to be updated. In the end, a single transfer model is returned, constructed from only the top‐scoring expressions.

## 3 Bioprocess Case Study

This study considers an in silico case study representing yeast fermentation for producing astaxanthin via glucose substrate consumption. The *source* model, in Equation (6), describes the evolution of biomass (X), substrate (S), and product (P) concentration, with the parameters: $\theta_{s}=\left(\mu_{m},K_{S},Y_{S},Y_{P},\beta \right)$, where $\mu_{m}=0.1\;h^{-1}$ is the maximum specific growth rate, $K_{S}=10g\;l^{-1}$ is the substrate half‐saturation constant, $Y_{S}=1g\;g^{-1}$ is the growth‐dependent substrate yield coefficient, while $Y_{P}=0.5g\;g^{-1}$ and β=0.003 $h^{-1}$ are the growth‐dependent and independent product yield coefficients, respectively.

(6a) $$\frac{dX}{dt}=\mu_{m}\frac{S}{S+K_{S}}X$$

(6b) $$\frac{dS}{dt}=-Y_{S}\;\mu_{m}\frac{S}{S+K_{S}}X$$

(6c) $$\frac{dP}{dt}=Y_{P}\mu_{m}\;\frac{S}{S+K_{S}}\cdot X+\beta \;X$$

In silico data for the *target* domain, representing a more productive strain, was generated using the ground‐truth model in Equation (7) (Vega‐Ramon et al. 2021). The target model parameters, $\theta_{t}=\left(\mu_{m},K_{S},\mu_{d},Y_{S},Y_{P},\beta ,k_{d}\right)$, share the same meanings as in the source model but with different values: $K_{S}=5\;g\;g^{-1}$, β=0.05 $h^{-1}$. New are a biomass decay constant, $\mu_{d}=0.1\;h^{-1}$, and product reversal rate under substrate‐limiting conditions, $k_{d}=0.07$ ${g^{-1}\;\text{l}\;\text{h}}^{-1}$ constant, while Equation (7) now uses Contois, rather than Monod kinetics, altogether reflecting physical differences between the source and target systems.

(7a) $$\frac{dX}{dt}=\mu_{m}\frac{S}{S+K_{S}X}\cdot X-\mu_{d}\;X$$

(7b) $$\frac{dS}{dt}=-Y_{S}\;\mu_{m}\frac{S}{S+K_{S}X}\cdot X$$

(7c) $$\frac{dP}{dt}=Y_{P}\mu_{m}\;\frac{S}{S+K_{S}X}\cdot X+\beta \;X-k_{d}X^{2}$$

Batch simulations each run for 144 h from initial conditions $y_{e}^{0}=\left(X_{0,e},S_{0,e},P_{0,e}\right)$. States $y_{i,e}=\left(X_{i,e},S_{i,e},P_{i,e}\right)$ were recorded every 14 h with normally distributed percentage noise with a standard deviation of 5% applied to the observations, representing typical bioprocess sampling frequencies and measurement noise (Bayer et al. 2020). Figure S1 in Section S2 shows how different the biomass, substrate, and product dynamics were between the source and target domains. Each case study starts with three experiments from three different initial conditions. To assess reproducibility and data sensitivity, the whole framework is re‐run ten times, starting from different sets of three experiments, generated from different random initial conditions over the range: $0.1\leq X\leq 0.5\;g\;l^{-1}$, $1\leq S\leq 10\;g\;l^{-1}$, and $P=0\;g\;l^{-1}$.

## 4 Results and Discussion

The robustness of the model structural transfer learning framework will be investigated in Section 4.1 under high prior knowledge conditions (i.e., high inductive bias) when there is a detailed but incorrect source model available, and in Section 4.2 under low prior knowledge conditions (i.e., low inductive bias) when only the general structure of the equations is known.

### 4.1 Scenarios With High Inductive Bias

Under high prior knowledge conditions, Equation (6) was used as the source model. Initially, in Section 4.1.1, only three initial experiments are generated for each of the 10 repetitions, further experiments are then added in Section 4.1.2 via MbDoE.

#### 4.1.1 Single Versus Multiple‐Passes of Model Structure Modification

A single‐pass approach first embeds multiple correction terms into the source model, which are then sparingly refined. Equation (8) shows for Step 1 in Figure 1 where twelve correction terms, φ, were automatically embedded into the source model structure. Within bioprocess kinetic modeling the specific growth rate term, μ, is often shared between expressions, therefore was treated as a fourth equation and a form of extra prior knowledge. Here $\phi_{j}^{\text{GT}}$ will denote the *simplest* canonical symbolic corrections when comparing the correction terms with the ground truth equations which depends on the choice of correction terms. Comparison with the target ground‐truth in Equation (7) indicates three key corrections—one for adapting the Monod to the Contois kinetics ($\phi_{2}^{\text{GT}}=X$), one for biomass decay ($\phi_{7}^{\text{GT}}=-\mu_{d}X$), and another for product reversal ($\phi_{12}^{\text{GT}}=-k_{d}X^{2}$), while other terms ideally vanish. In Step 1, across ten random repetitions, $\varphi_{7}$ and $\varphi_{12}$ were identified with 90% success and $\varphi_{2}$ was identified with 40% success, while $\varphi_{3}$ and $\varphi_{4}$ were identified 30% and 10% of the time, respectively. This variation is not overly problematic since should $\phi_{7}^{\text{GT}}=-\mu_{d}X$ and $\phi_{12}^{\text{GT}}=-k_{d}X^{2}$ be proposed, SR can later propose either $\phi_{3}^{\text{GT}}=\left(S+K_{S}X-K_{S}\right)/S$ or $\phi_{4}^{\text{GT}}=\left(S+K_{S}\right)/\left(S+K_{S}X\right)$ as equally valid corrections; though these are not the *simplest* symbolic corrections.

(8a) $$\mu ={\varphi_{4}\mu }_{m}\frac{S+\varphi_{1}}{\varphi_{3}S+{\varphi_{2}K}_{S}}+\varphi_{5}$$

(8b) $$\frac{dX}{dt}=\varphi_{6}\mu X+\varphi_{7}$$

(8c) $$\frac{dS}{dt}=-\varphi_{8}Y_{S}\;\mu X+\varphi_{9}$$

(8d) $$\frac{dP}{dt}={\varphi_{11}Y}_{P}\mu X+\varphi_{10}\beta X+\varphi_{12}$$

In Step 2, the input features (i.e., X, S or P) most important for constructing the symbolic form, ϕ, of the correction terms, φ, were identified. The canonical corrections are all based on X, but noncanonical corrections may require S. In Step 2, P was eliminated 100% of the time, and S was correctly handled 80% of the time. However, determining whether S should remain or be eliminated was not always possible until P was removed, showing how an iterative approach to feature pruning was necessary.

**Figure 4:**
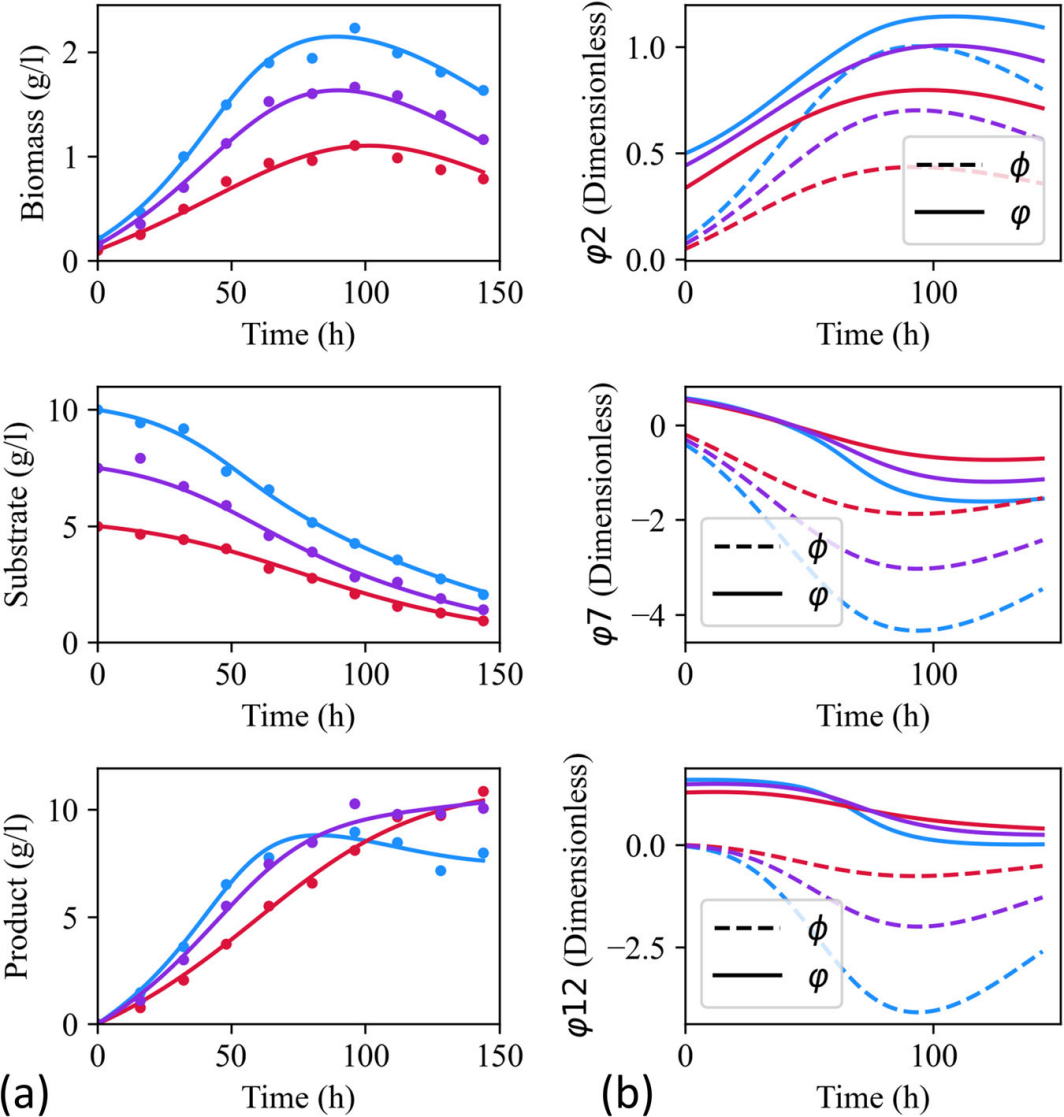
An example showing the fitted process measurements (a) and the fitted correction term profiles ($\hat{\varphi }$) compared with the canonical symbolic correction term profiles (ϕ) (b) when Step 1 has identified $\varphi_{2}$, $\varphi_{7}$, and $\varphi_{12}$ as being the locations of the corrections. The three experiments colored blue, red, and purple.

Even when the measured state variables are fitted well in Figure 4a, some offset can exist in Figure 4b between the fitted correction terms, $\hat{\varphi }$, and the canonical symbolic corrections, ϕ. This was because $\varphi_{2}$, $\varphi_{7}$ and $\varphi_{12}$ are structurally nonidentifiable when estimated simultaneously with the yield coefficients $Y_{S}$ and $Y_{P}$. Section 4.3 will discuss further some ways extra constraints or prior knowledge can be incorporated to address this problem.

After carrying out Steps 1–4, across the 10 random repetitions, the overall success rate was only 10%. Equation (9) shows an example of an incorrect target model, where $\phi_{2}=X+0.40$, but $\phi_{2}^{\text{GT}}=X$ has a sparse representation within $\phi_{2}^{\prime }$ due to the almost systematic offset shown in Figure 4b. If sparsity is promoted by penalizing the magnitude of the kinetic parameters, θ, during Step 4, the success rate increases from 10% to 50%. However, Equation (9c) illustrates a case sparse finetuning cannot correct where $\phi_{12}^{\text{GT}}=-k_{d}X^{2}$ was instead $\phi_{12}=1.88S$ and so product reversal under substrate‐limiting conditions was falsely attributed to a product accumulation term that simply diminished towards the end of the batch.

(9a) $$\frac{dX}{dt}=X\mu +1.03S-1.93X$$

(9b) $$\frac{dS}{dt}=-Y_{s}\mu X$$

(9c) $$\frac{dP}{dt}=Y_{P}\mu X+\beta X+1.88S$$

(9d) $$\mu =\mu_{m}\frac{S}{S+K_{s}\left(X+0.40\right)}$$

**Figure 5:**
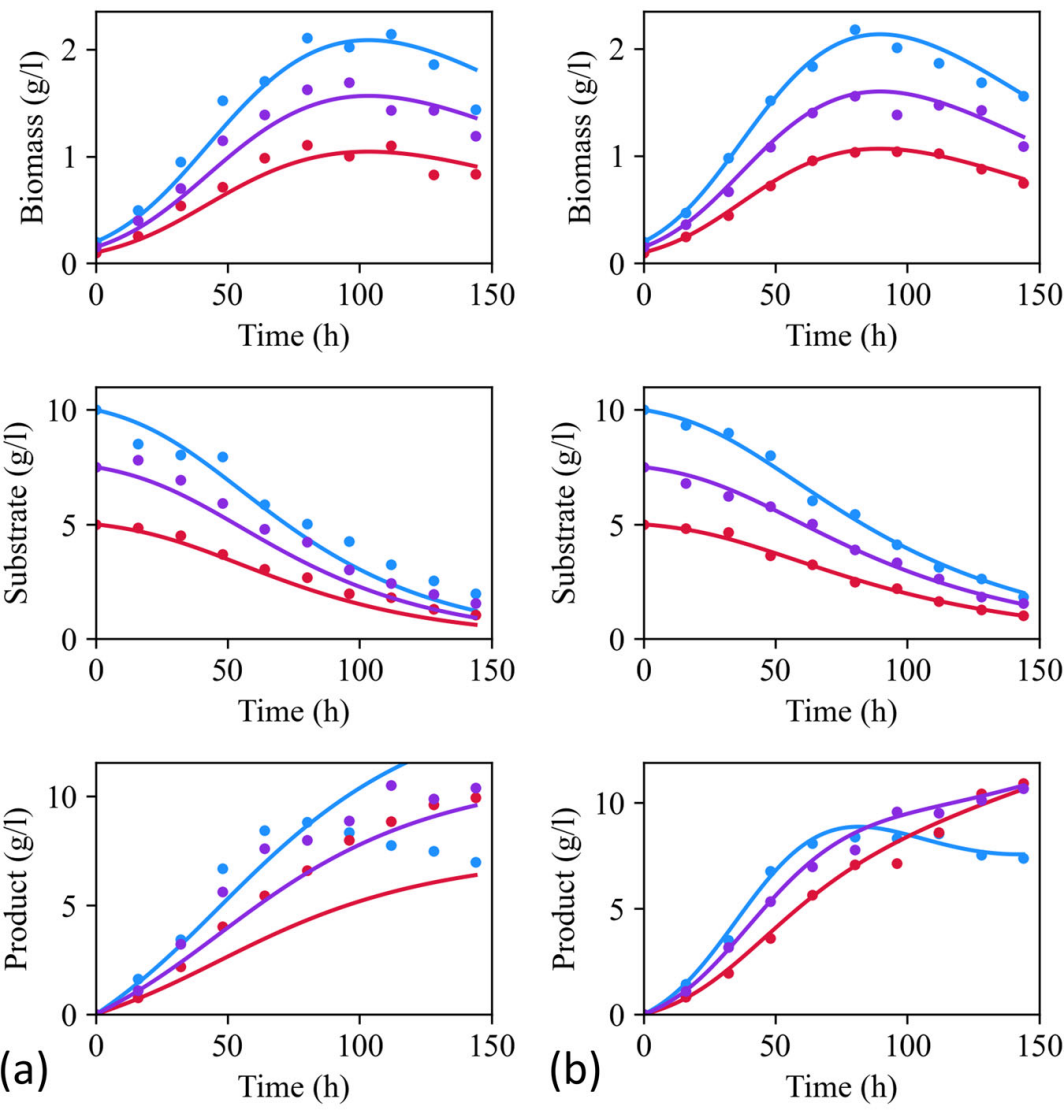
Comparison of the model (Equation 9) fitting three initial experiments after one pass (a) and after three passes (b) of model structure modification. The initial conditions for these experiments were (X=0.1, S=5, P=0), (X=0.15, S=7.5, P=0), and (X=0.2, S=10, P=0), where X, S, and P denote biomass, substrate, and product concentration ($g\;l^{-1}$).

Now, the equations are returned for further passes to assess whether partially corrected equations can be fully adapted to the target domain. No further passes are required once there are no correction terms with a variance, $\tilde{\varphi }$, greater than, the threshold, $V_{\min}$. Compared with a single pass, the success rate improves from 50% to 80%. Following the example in Equation (9), the mean absolute percentage error (MAPE) of fitting improved from 17% in Figure 5a to 6.6% in Figure 5b once of multiple passes discovered the ground‐truth. Meanwhile, the average MAPE of prediction across all 10 random repetitions improved from 23% to 10.7%. Therefore, an average of two extra passes improved the framework's reliability. The equations failed to update after further passes, indicating convergence. However, failing to locate the simplest corrections in early passes can sometimes make it difficult to maintain accurate yet parsimonious structures in later passes.

#### 4.1.2 Model‐Based Design of Experiments

When only a few noisy experiments are available, multiple candidate structures may explain the data similarly well. MbDoE identifies experimental conditions for discriminating among those candidates. For example, in the beginning, X and $\theta_{1}-\theta_{2}S$, where $\theta_{1}$ and $\theta_{2}$ are constants, could be used almost interchangeably in the model structure due to the large fraction of substrate that is converted to biomass relative to product (i.e., S→X). Only once enough experiments were designed was $X\approx \theta_{1}-\theta_{2}S$ recognized as incorrect. Consequently, the product‐reversal term ($\phi_{12}^{\text{GT}}=-k_{d}X^{2}$) was typically discovered last when it was finally elucidated that $\phi_{12}=f\left(X\right)$ not $\phi_{12}=f\left(X,S\right)$ was the simplest correction, highlighting how separating the causal relationship can be difficult when state variables are strongly correlated. In Figure 5, given three initial experiments, the average MAPE was 11.2% with a standard deviation of 3.1% across the 10 repetitions. This decreased to 5.3% with a standard deviation of 0.1% after 13 new MbDoE experiments. Eight out of 10 runs achieved an MAPE of 6.0% by the fourth MbDoE iteration, at which point all the runs discovered the ground truth equation structures for the target domain.

### 4.2 Scenarios With Low Prior Knowledge

In contrast to the previous case, which provided greater structural information on the form of the rate expressions, only minimal, generic understanding is now supplied: biomass growth, substrate consumption, and product formation are each proportional to biomass concentration. Such established knowledge requires no experimental validation. However, with no process‐specific information, applying the framework to elucidate the biochemical reaction kinetics is now more challenging—akin to investigating a wholly new bioprocess, such as a novel strain.

#### 4.2.1 Single Versus Multiple‐Passes of Model Structure Modification

Eight correction terms, φ, were initially embedded into the simplified source model structure to represent new or revised kinetics, as shown in Equation (10). The principal prior knowledge here is the understanding that there exists a growth‐dependent term shared between the state equations.

(10a) $$\frac{dX}{dt}=\varphi_{3}\mu X+\varphi_{4}$$

(10b) $$\frac{dS}{dt}=-\varphi_{5}Y_{S}\;\mu X+\varphi_{6}$$

(10c) $$\frac{dP}{dt}={\varphi_{7}Y}_{P}\mu X+\varphi_{8}$$

(10d) $$\mu ={\varphi_{1}\mu }_{m}S+\varphi_{2}$$

Comparison between Equation (10) and the ground truth target model in Equation (7) shows that of the eight corrections, $\phi_{1}^{\text{GT}}=1/({S+K}_{S}X)$, $\phi_{4}^{\text{GT}}=-\mu_{d}X$, and $\phi_{8}^{\text{GT}}=\beta X-k_{d}X^{2}$ are the canonical symbolic corrections. In the first pass, $\varphi_{4}$ and $\varphi_{8}$ were accurately located with 90% accuracy, while $\varphi_{1}$ was correctly located only 30% of the time, with $\varphi_{3}$ located in 70% of cases. Next, the symbolic form, ϕ, was built as a function of the fitted states, $\hat{y}$, and substituted for sparse parameter fine‐tuning. Equation (11) illustrates where $\varphi_{3}$, $\varphi_{4}$, and $\varphi_{8}$ were located, showing how endogenous biomass decay (−2.38X) and growth‐independent product accumulation (1.13X) were discovered. However, in this low prior knowledge scenario, the source model structure was rarely fully corrected in a single pass. A common mistake was failing to globally correct the specific growth rate, μ, as seen in Equation (11b), where μ was only modified locally in the biomass growth equation. Despite this, the overall success rate was 40%.

(11a) $$\frac{dX}{dt}=\mu X\left(\frac{S}{0.21S+X}+0.23\right)-2.38X$$

(11b) $$\frac{dS}{dt}=-Y_{s}\mu X$$

(11c) $$\frac{dP}{dt}=Y_{P}\mu X+1.13X+0.76S$$

(11d) $$\mu =\mu_{m}S$$

The equations were then returned for further passes. Once endogenous biomass decay and growth‐independent product accumulation were identified in the first pass, the number of necessary terms, $n_{\varphi }$, with variance, $\tilde{\varphi }$, less than the threshold, $V_{\min}$, decreased and further corrections became more targeted. The success rate increased from 40% to 50% over an average of four passes, though the improvement was smaller compared to the high inductive bias case (i.e., 50%–80%). This was because SR attempted to construct more complex correction, terms, ϕ, in the first pass making mistakes harder to correct in later passes. Thus, a conservative approach (i.e., a lower $C_{\max}$) was beneficial (i.e., improved from 50% to 60%).

#### 4.2.2 Model‐Based Design of Experiments

**Figure 6:**
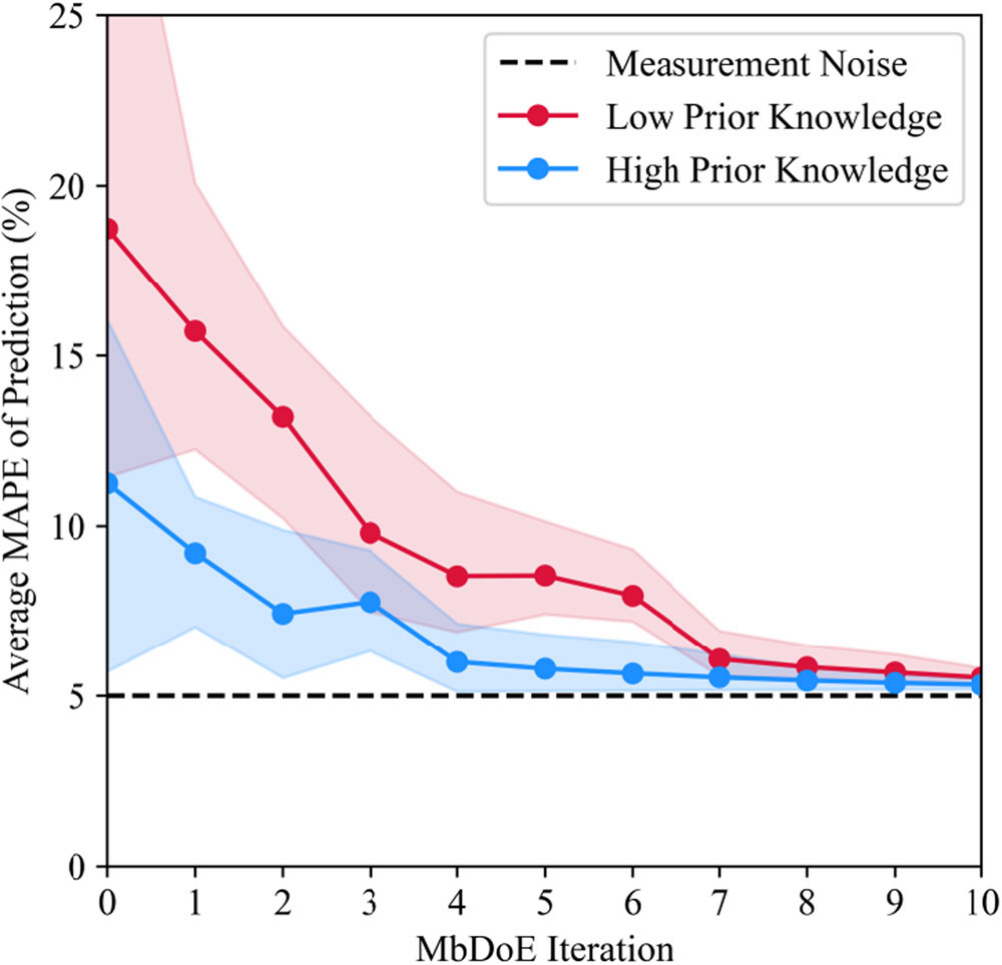
Comparison between low and high prior knowledge scenarios, showing the mean‐absolute percentage error (MAPE) of prediction over successive model‐based design of experiment (MbDoE) iterations. The shaded region denotes three standard deviations in the MAPE over the 10 repetitions of the framework from different random initial datasets, while the markers denote the average MAPE. Here multiple passes of model structure modification were allowed.

Integrating the same transfer learning approach into MbDoE again improved accuracy. Initially, in Figure 6, the MAPE was 18.7% reducing to 5.5% by the final iteration and the 13th experiment. Eight out of 10 runs reached a MAPE of 6.2% by the seventh iteration, at which point 100% of the runs had identified the ground truth equation structures for the target domain—more experiments than in the high‐prior knowledge scenario due to more of the dynamics needing to be discovered. However, this will be highly case‐by‐case. The larger initial variation with a standard deviation of 5.9% rather than 3.1%, seen graphically in the width of the shaded region in Figure 6, also underscores the greater sensitivity to initial experimental design when less prior knowledge is available.

### 4.3 Tuning the Framework for General Application

#### 4.3.1 Source‐Target Domain Similarity

It was shown in the previous section that incorporating prior physical knowledge reduces experimental burden. However, transfer learning is only effective when the source and target systems are sufficiently similar—something seldom known a priori. This is as true here as it is in traditional transfer learning (Ehrig et al. 2024; Tahir et al. 2024). Therefore, in this section, we provide practical guidance to assess whether mechanistic model structure transfer learning is likely to be effective before performing further experiments.

First, we recommend comparing the adapted model structure to the original: if substantial, unintuitive changes are required to fit the target system, the source and target models are likely incompatible. Second, we suggest starting with the simplest source model to identify key correction terms, then comparing the learned equations with those from higher prior knowledge models. For illustration, Equations (12a) and (12b) reproduce the source models for biomass growth under low and high prior knowledge, respectively, while Equation (12c) incorrectly adds a substrate inhibition term, $K_{I}/S$, and a product toxicity term, $P/(P+K_{P})$. Here, we would recommend progressing from Equations (12a) to (12b) to (12c) sequentially as candidate source models.

(12a) $$\frac{dX}{dt}=\mu X$$

(12b) $$\frac{dX}{dt}=\mu_{m}\frac{S}{S+K_{S}}X$$

(12c) $$\frac{dX}{dt}=\mu_{m}\frac{S}{S+K_{S}+\frac{K_{I}}{S}}\cdot X-\mu_{d}\;X\frac{P}{P+K_{P}}$$

The earlier results showed overlap in the discovered correction terms across the low and high prior knowledge scenarios (e.g., endogenous biomass decay, substrate‐correlated product accumulation), while similar terms in the high prior knowledge source model were recovered even when starting from the low prior source model (e.g., growth‐independent product accumulation). Since equivalent features were discovered, this suggests that the high prior knowledge model was sufficiently similar to the target system. Conversely, no corrections resembling the substrate inhibition or product toxicity terms in Equation (12c) were discovered. When multiple levels of source models, such as those shown in Equation (12), are unavailable, an existing model can be decomposed by sequentially stripping away assumptions.

#### 4.3.2 Mutual Correlation of Correction Terms

Mutual correlation between correction terms can occur when there are multiple solutions to the correction terms and parameters that fit the data equally well, akin to structural non‐identifiability. To generalize the example that occurred in Section 4.1, consider the general bioprocess kinetics equation in Equation (13), where there is a growth‐dependent term, $\varphi_{\mathrm{GD}}\left(y\right)$, and a growth‐independent term, $\varphi_{\mathrm{GI}}\left(y\right)$ (e.g., biomass decay, product reversal, or substrate consumption for maintenance).

(13) $$\frac{dy}{dt}=\mu \cdot \varphi_{\mathrm{GD}}\left(y\right)+\varphi_{\mathrm{GI}}\left(y\right)$$

Mathematically, the overall rate would be unchanged by increasing $\varphi_{\mathrm{GD}}\left(y\right)$ while decreasing $\varphi_{\mathrm{GI}}\left(y\right)$, or vice versa. Hence, product reversal $\phi_{12}=-k_{d}X^{2}$ could often be approximated by $\phi_{12}^{\prime }\propto S$, even though biomass and substrate were inversely correlated (i.e., S→X). Similar issues can arise whenever one (bio)chemical species reacts to form another (e.g., A+2B→C). MbDoE can propose experiments that break these correlations, but at the cost of wasting experiments that would have been unnecessary if only the physics had been considered. In batch bioprocesses, particularly during the early stages, growth‐independent terms are generally negligible (i.e., $\varphi_{\mathrm{GI}}\left(y\right)\to 0$ as t→0). This is because the initial phase is dominated by exponential cell proliferation, where growth‐associated kinetics prevail. This knowledge can be included by regularization during correction term fitting by penalizing the sum of the terms corresponding to $\varphi_{\mathrm{GI}}\left(y\right)$ from deviating from zero around the start of the batch simulations. With this, it was possible, starting from the high prior knowledge source model in Eq. 6, to reliably identify the product reversal term without conducting further experiments.

#### 4.3.3 Hyperparameter Selection

There are two key hyperparameters in the model structure transfer learning algorithm: (1) the maximum number of correction terms, $U_{\max}$, that can be constructed simultaneously, and (2) the maximum complexity, $C_{\max}$, that can be added or removed by cancelation from the source model equations in one pass of model structure modification. When applying the framework to other problems, the first key hyperparameter, $U_{\max}$, should not exceed the number of dynamic state variables to avoid structural non‐identifiability. Since in the case study explored in this study, there were only three dynamic state variables (i.e., X, S, and P), $U_{\max}=3$. Conversely, if $U_{\max}$ is set too low, only a few locations can be corrected simultaneously, causing nonlinear model‐process mismatches to be lumped into those locations, resulting in highly nonlinear and less interpretable correction expressions. This is where the second key hyperparameter, $C_{\max}$, can be important. The larger $C_{\max}$ is, the more complex and aggressive the updates will be to the source model, which can be more difficult to backtrack. The smaller $C_{\max}$ is, the simpler and more conservative the updates, which may mean more passes of model structure modification are required but terms can always be added or refined if corrections were missed in the first iteration. Low prior knowledge scenarios will generally suit a more a conservative approach to first fill in the main relationships before later refinement. The other hyperparameters include the correction term depth, $D_{\max}$, regularization penalty coefficients, $\lambda_{1}$ and $\lambda_{2}$, and the variance threshold for controlling whether to continue improving the model, $V_{\min}$. These were determined by independent calibration. Overall, while the number of hyperparameters remains a limitation of the framework, they provide substantial flexibility for human‐machine interaction, which is essential since in practice it often comes down to human judgment as to how complex the model should be for a new process. A fundamentally complex system may require a complex model. Here, hybrid models offer more fitting flexibility, since suitable corrections can only ever be approximated by parsimonious symbolic expressions. In such cases, there will be a compromise between fitting accuracy and interpretability.

## 5 Conclusion

In conclusion, this study introduces a novel structural transfer learning framework that integrates SR with ANN feature attribution to improve the accuracy of biochemical kinetic models. Unlike traditional black‐box‐based transfer learning approaches that focus only on parameter adjustments, our method modifies the structure of kinetic models, enabling the discovery of interpretable differential equations for a newly investigated process. Through a series of in silico case studies, we demonstrated the robustness of the proposed framework under low‐sample frequency, noisy data conditions with strong inductive bias, or limited prior knowledge, showcasing its capacity to make targeted corrections or generate new interpretable expressions whenever necessary. The approach was also demonstrated to synergize well with model‐based design of experiments for discrimination between possible transfer model candidates to minimize experimental resources. Although the method involves several hyperparameters, this flexibility is necessary for human‐machine interaction, allowing human judgment to guide the model's complexity for new processes. With the framework also capable of approximating time‐varying kinetics, avenues for future work exist in putting this to the test and extending the framework to history‐dependent processes. Overall, this study advances automated model discovery by providing a more interpretable and physically meaningful approach to transfer learning, which can accelerate new bioprocess development with potential to also support the general chemical and formulation industry.

## Nomenclature

Symbol: Meaning Units Equation
y
: concentrations of (bio)chemical species ($g\;l^{-1}$), —
t
: time from start of batch experiment (h), —
θ
: vector of kinetic parameters (—), —
$f_{s}\left(y,\theta_{s}\right)$
: source model equations (—), —
$\theta_{s}$
: vector of source kinetic parameters (—), —
$f_{t}\left(y,\theta_{t}\right)$
: transfer model equations (—), —
$\theta_{t}$
: vector of transfer kinetic parameters (—), —
π
: vector of input feature weightings (—), —
$\hat{y}$
: vector of predicted state variables (—), —
$\hat{\varphi }$
: vector of fitted correction terms (—), Equation (2b)
ϕ
: vector of symbolic correction terms (—), —
$f_{g}\left(y,\theta_{t}\right)$
: ground truth equations for target domain (—), —
ω
: vector of ANN weights and biases (—), —
$L_{\text{ANN}}$
: loss function for fitting correction terms (—), Equation (2a)
${\hat{y}}_{i,e}$
: $\hat{y}$ at time index i and experiment e (—), Equation (2d)
$y_{i,e}$
: y at time index i and experiment e (—), —
i
: measurement time index (—), —
e
: experiment number index (—), —
$n_{\varphi }$
: total current number of correction terms (dimensionless), —
$n_{i}$
: total number of samples per experiment (dimensionless), —
$n_{e}$
: total current number of experiments (dimensionless), —
$t_{i}$
: time at measurement time index i (dimensionless), —
$y_{0}^{e}$
: initial conditions for experiment e (—), —
$U_{\max}$
: hyperparameter controlling the maximum number of simultaneous correction terms (dimensionless), —
$V_{\min}$
: hyperparameter controlling the minimum variance permitted for correction terms (—), —
$L_{\text{SR}}$
: loss function for symbolic regression (—), Equation (3)
${\hat{\varphi }}_{i,e}$
: fitted correction at time i and experiment e (—), —
$C_{\max}$
: maximum complexity of symbolic correction (—), —
z
: general experiment design variable (—), —
J(z)
: general objective function to design new experiment for MbDoE (—), —
$J\left(y_{0}\right)$
: objective function to design new experiment in the case study as a function of initial conditions (—), Equation (S2)
$f_{t}^{m}\left(y,\theta_{t}\right)$
: transfer model equations candidate m (—), —
m
: model candidate index (—), —
X
: biomass concentration ($g\;l^{-1}$), —
S
: substrate concentration ($g\;l^{-1}$), —
P
: product concentration ($g\;l^{-1}$), —
$\mu_{m}$
: maximum specific growth rate ($h^{-1}$), —
$K_{S}$
: substrate half‐saturation constant ($g\;l^{-1}$), —
$Y_{S}$
: growth‐dependent substrate yield coefficient ($g\;g^{-1}$), —
$Y_{P}$
: growth‐dependent product yield coefficient ($g\;g^{-1}$, —
β
: growth‐independent product yield coefficient ($h^{-1}$), —
$\mu_{d}$
: endogenous biomass decay constant ($h^{-1}$), —
$k_{d}$
: product reversal rate (${g^{-1}\text{l h}}^{-1}$), —
$\phi^{\text{GT}}$
: simplest canonical symbolic corrections by comparison with the ground truth equations (—), —
μ
: specific growth rate term ($h^{-1}$)
$\varphi_{\mathrm{GD}}\left(y\right)$
: growth dependent kinetics (g)
$\varphi_{\mathrm{GI}}\left(y\right)$
: growth independent kinetics (${\text{g h}}^{-1}$)
$D_{\max}$
: hyperparameter controlling maximum depth into the expressions' tree representation corrections can be embedded (—), —
$\lambda_{1}$
: penalty weight for correction Jacobian (—), Equation (2e)
$\lambda_{2}$
: penalty weight for number of correction terms (—), Equation (2e)
j
: correction term index (—), —
$\varphi_{j}$
: correction term j (—), —
${\bar{\varphi }}_{j}$
: mean of correction term $\varphi_{j}$ (—), Equation (3a)
${\tilde{\varphi }}_{j}$
: variance of correction term $\varphi_{j}$ (—), Equation (3b)
$Y_{0}$
: space of allowable initial conditions $y_{0}$ (—), —
$n_{v}$
: number of input features (dimensionless), —
$n_{i}$
: number of sample intervals per experiment (dimensionless), —
$n_{j}$
: current number of correction terms (dimensionless), —
$n_{e}$
: number of experiments in current data set (dimensionless), —
$n_{m}$
: number of overall model candidates (dimensionless), —
v
: input feature for symbolic regression index (—), —
$y_{0}$
: vector of initial conditions (—), —
$y_{0}^{e}$
: vector of initial conditions for experiment e
${\text{IG}}_{v,i,e}^{j}\left(y\right)$
: integrated gradient attribution function (—), Equation (S3a)
$\sigma_{v}^{j}$
: aggregated feature attributions for correction term j and feature v (—), Equation (S3b)
$\pi_{v}^{j}$
: aggregated, normalized feature attributions for correction term j and feature v (—), Equation (S3c)
k
: index for symbolic correction term candidate (—), —
$\phi_{j,k}$
: symbolic correction for $\varphi_{j}$ candidate k
$S_{j,k}$
: score to balance complexity versus fitting for $\phi_{j,k}$ (—), —
$L_{j,k}^{\text{SR}}$
: loss function for $\phi_{j,k}$ (—), Equation (S4a)
$C_{j,k}$
: complexity of $\phi_{j,k}$ (—), —
$L_{\text{NR}}$
: loss function for final parameter finetuning (—), Equation (S4a)
P(y,ω)
: penalty for ANN correction term fitting (—), Equation (2e)

## Author Contributions

**Alexander W. Rogers:** conceptualization, formal analysis, investigation, methodology, software, visualization, writing – original draft, writing – review and editing. **Fernando Vega‐Ramon:** conceptualization, software, writing – review and editing. **Amanda Lane:** project administration, supervision, writing – review and editing. **Philip Martin:** funding acquisition, project administration, supervision. **Dongda Zhang:** conceptualization, funding acquisition, investigation, methodology, supervision, writing – review and editing.

## Supporting information

Revised Supplementary ‐ Model Structural Transfer Learning.

## Data Availability

The data that support the findings of this study are available from the corresponding author upon reasonable request.
