# Supplementary material for: Interpretable‐AI‐Based Model Structural Transfer Learning to Accelerate Bioprocess Model Construction
Source: Biotechnol Bioeng. 2025 Jul 18;122(10):2819–31. doi: 10.1002/bit.70026 (PMC12417796; doi:10.1002/bit.70026)
Supplement: Supplementary file 1 — Revised Supplementary ‐ Model Structural Transfer Learning. [file BIT-122-2819-s001.docx]

# S1. Mean and Variance Definitions

Each iteration, the least important corrections are defined as those with the smallest variance over the input domain. In Eq. S1a and S1b, where $\bar{\varphi}_{j}$ and $\tilde{\varphi}_{j}$ are the mean and variance, respectively, over the input domain.

$$\begin{aligned} \bar{\varphi}_{j}=\frac{1}{n_{i}n_{e}}\sum_{i=1}^{n_{i}} \sum_{e=1}^{n_{e}} \varphi_{j}\left( \boldsymbol{y}_{i,e}\boldsymbol{,\omega} \right)\#\left( S1a \right) \end{aligned}$$

$$\begin{aligned} \tilde{\varphi}_{j}=\frac{1}{n_{i}n_{e}}\sum_{i=1}^{n_{i}} \sum_{e=1}^{n_{e}} \left( \varphi_{j}\left( \boldsymbol{y}_{i,e}\boldsymbol{,\omega} \right)-\bar{\varphi}_{j} \right)^{2}\#\left( S1b \right) \end{aligned}$$

# S2. Comparison Between High Prior Knowledge Source and Target Domain

Fig. S1 shows how the biomass growth, substrate consumption and product accumulation profiles differ significantly between the source and target domain.


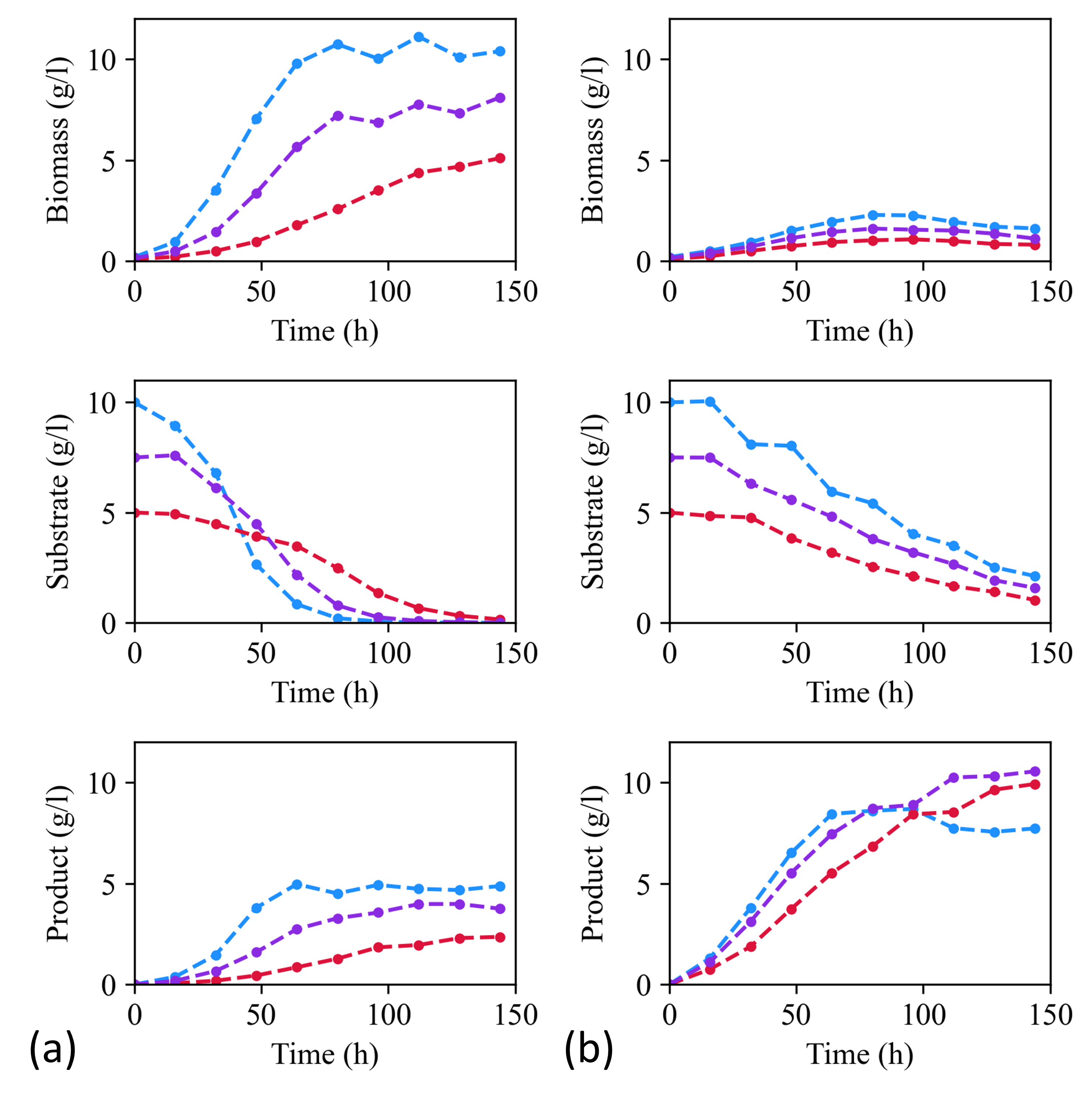


Fig. S1: The ground-truth process trajectories for (a) source domain when high but incorrect prior knowledge was available and (b) target domain. The initial conditions for these three experiments were ($X=0.1$, $S=5$, $P=0$), ($X=0.15$, $S=7.5$, $P=0$) and ($X=0.2$, $S=10$, $P=0$), where $X$, $S$ and $P$ denote biomass, substrate and product concentration ($g l^{-1}$).

**S3. Calculation of Feature Attributions**

Defined in Eq. S2a, the gradient of each fitted functional correction, $\hat{\varphi}$, with respect to each input feature, $y_{v}$, was integrated along the fitted process state trajectory, ${\hat{\boldsymbol{y}}}_{i,e}$, from the initial condition, $\boldsymbol{y}_{e}^{0}$, for each experiment, $e$. Global feature importance is retrieved by aggregating local feature attributions as the mean absolute value (Eq. S2b), then normalised (Eq. S2c).

$$\begin{aligned} \mathrm{IG}_{v,i,e}^{j}\left( \boldsymbol{y} \right)=\int_{0}^{t_{i}} \frac{\partial\hat{\varphi}_{j}}{\partial y_{v}}\left( {\hat{\boldsymbol{y}}}_{t,e} \right)dt\#\left( S2a \right) \end{aligned}$$

$$\begin{aligned} \sigma_{v}^{j}\mathbf{=}\frac{1}{n_{i}n_{e}}\sum_{i=1}^{n_{i}} \sum_{e=1}^{n_{e}} \left| \mathrm{IG}_{v,i,e}^{j}\left( \boldsymbol{y} \right) \right|\boldsymbol{\#}\left( S2b \right) \end{aligned}$$

$$\begin{aligned} \pi_{v}^{j}\mathbf{=}\frac{\sigma_{v}^{j}}{\left\| \boldsymbol{\sigma}^{j} \right\|_{1}}\boldsymbol{\#}\left( S2c \right) \end{aligned}$$

The resulting aggregated, normalised attributions, $0\leq\pi_{v}^{j}\boldsymbol{\leq}1$, represent the relative contribution of each feature, $y_{v}$, to each fitted function correction, $\hat{\varphi}$. These aggregated, normalised attributions are used to weight the features during symbolic regression.

**S4. Supplementary Section S4etails on Symbolic Regression**

In this study, genetic programming (GP) by tournament selection promoted and mutated candidates from a large population of expressions represented by directed acyclic graphs, using *SymbolicRegression* (Cranmer, 2023), customised to facilitate feature weighting.


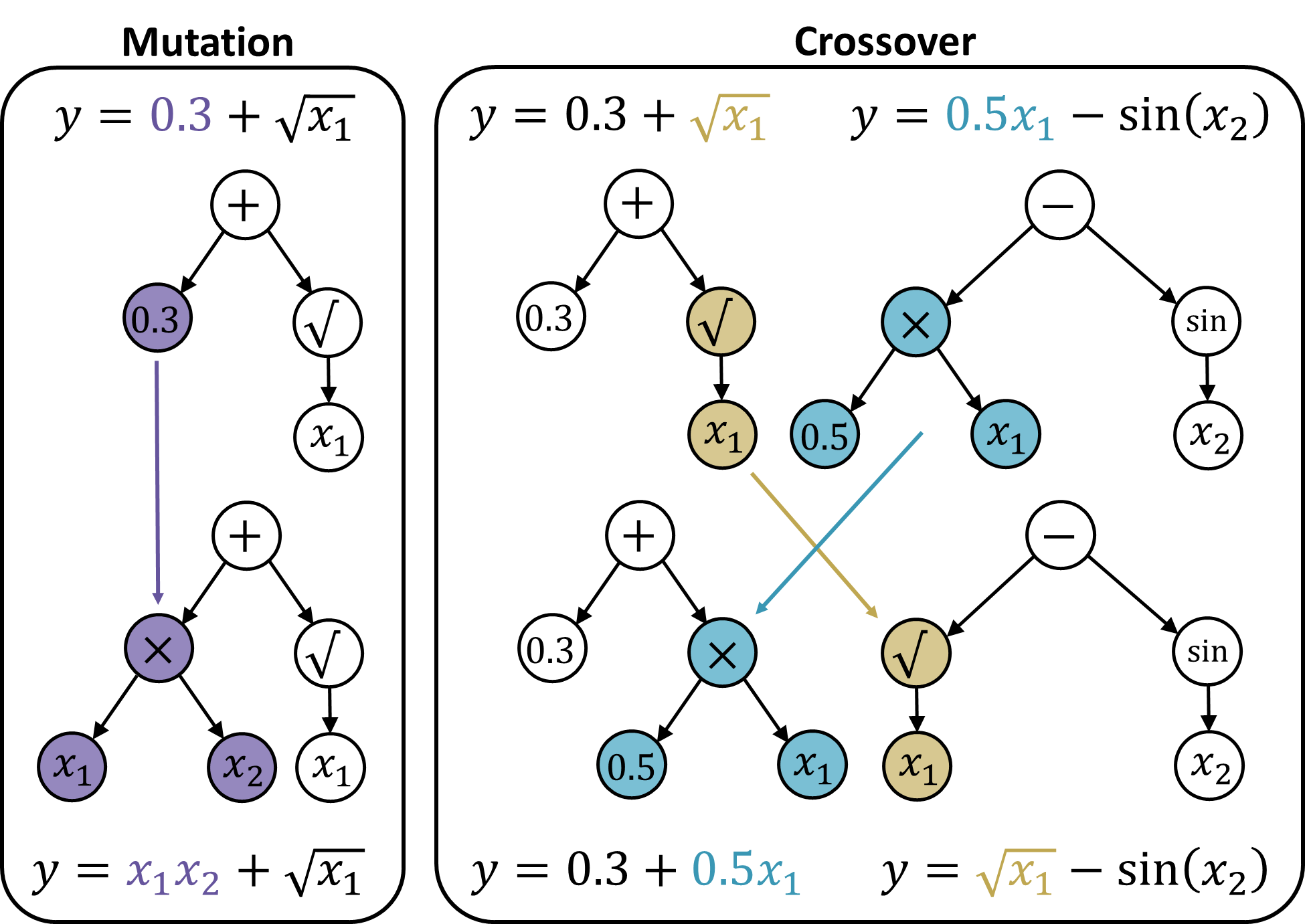


Fig. S2: Illustration of mutation and crossover events for SR by genetic programming.

Initially, 40 parallel populations of 100 individuals are generated randomly. During each tournament, the fitness of each expression is evaluated using Eq. S3a which is the mean-square-error between the value predicted by each expression, $\phi$, given the fitted concentrations, ${\hat{\boldsymbol{y}}}_{i,e}$, and the values of the fitted corrections, $\hat{\varphi}_{i,e}$ averaged over each time point, $i$, experiment, $e$. During each tournament, a random subsample of individuals is drawn with the fittest candidates more likely to be selected and carried forward for mutation and crossover as illustrated in Fig. S2. The winners replace the oldest members of the population. *SymbolicRegression* (Cranmer, 2023) integrates this GP within an *evolve-simplify-optimise* loop so that after a set number of tournaments and mutations, expressions are simplified using algebraic equivalences and the parameters refined through local-gradient-based optimisation.

$$\begin{aligned} \mathcal{L}_{\mathrm{SR}}\left( \phi, \boldsymbol{\theta} \right)=\frac{1}{n_{i}n_{e}}\sum_{i=1}^{n_{i}} \sum_{e=1}^{n_{e}} \left( \phi\left( {\hat{\boldsymbol{y}}}_{i,e}\boldsymbol{,\theta} \right)-\hat{\varphi}_{i,e} \right)^{2}\#\left( S3a \right) \end{aligned}$$

$$\begin{aligned} S_{k}=-\frac{\log\left( \mathcal{L}_{k+1}^{\mathrm{SR}} \right)-\log\left( \mathcal{L}_{k}^{\mathrm{SR}} \right)}{\mathcal{C}_{k+1}-\mathcal{C}_{k}}\#\left( S3b \right) \end{aligned}$$

At the end of SR, the fittest individuals at each complexity level, $\mathcal{C}_{k}$, are arranged on a Pareto front such that $\mathcal{C}_{k}<\mathcal{C}_{k+1}$. Each candidate expression, $k$, is given a score, $S_{k}$, using the negated derivative in their log-loss, $\log\mathcal{L}_{k}^{\mathrm{SR}}$, with respect to complexity, $\mathcal{C}_{k}$, as shown in Eq. S4b (Cranmer, 2023). Expressions with a higher score, $S_{k}$, will represent elbow points on the Pareto front thereby balancing fitting accuracy and complexity.

**S5. Final Target Model Kinetic Model Parameter Finetuning**

Eq. S4 displays the optimisation problem solved for final parameter finetuning, where all symbols have the same meaning as in the main text of this manuscript. Sparsity is promoted in the target model kinetic parameters by the $\lambda$-weighted penalty, $\left\| \boldsymbol{\theta}_{t} \right\|_{2}$, that penalises the absolute magnitude of the parameter vector.

$$\begin{aligned} \min_{\boldsymbol{\theta}_{t}} \mathcal{L}_{\mathrm{NR}}\left( \boldsymbol{\theta}_{t} \right)=\min_{\boldsymbol{\theta}}\frac{1}{n_{i}n_{e}}\sum_{i=1}^{n_{i}} \sum_{e=1}^{n_{e}} \left\| {\hat{\boldsymbol{y}}}_{i,e}\left( \boldsymbol{\theta}_{t} \right)\mathbf{-}\boldsymbol{y}_{i,e} \right\|_{2}+\lambda_{3}\times\left\| \boldsymbol{\theta}_{t} \right\|_{2}\#\left( S4a \right) \end{aligned}$$

$$s.t.$$

$$\begin{aligned} {\hat{\boldsymbol{y}}}_{i,e}\left( \boldsymbol{\theta}_{t} \right)\mathbf{=}\boldsymbol{y}_{0}^{e}\mathbf{+}\int_{0}^{t_{i}} f_{t}\left( \boldsymbol{y}\mathbf{,}\boldsymbol{\theta}_{t} \right)dt\#\left( S4b \right) \end{aligned}$$

Note that this optimisation problem is solved while still in the nondimensionalised time-state space where the ground truth kinetic model parameters all have similar magnitude. This way any additional parameters that should not be present can be pushed towards zero then removed and then optimisation problem resolved without the penalty (i.e., $\lambda_{3}=0$). For this optimisation problem Adaptive Moment Estimation (ADAM) was used for efficiency since the parameter values should not need adjusting drastically but global optimisation approaches may provide better guarantees. The recommended approach in that case would be a two-step approach: first global optimisation (e.g., differential evolution or Bayesian optimisation) to find the rough location of the global optimum, followed by gradient based finetuning.

**S6. Hyperparameter Selection and Calibration Equations**

Seven hyperparameters (i.e., $\mathcal{D}_{\max}$, $\lambda_{1}, \lambda_{2}$ and $\lambda_{3}$, $\mathcal{U}_{\max}$, $\mathcal{C}_{\max}$ and $\mathcal{V}_{\min}$) are involved in the elucidation of the corrections. Table S1 shows the values used in the case study in this research.

Table S1: Hyperparameter values used in the case study in this research.

| Hyperparameter | Value Used in Case Studies |
| --- | --- |
| $\mathcal{D}_{\max}$ | 3 |
| $\lambda_{1}$ | 0.1 |
| $\lambda_{2}$ | 0.1 |
| $\lambda_{3}$ | 0.05 |
| $\mathcal{U}_{\max}$ | 2 |
| $\mathcal{C}_{\max}$ | 10 |
| $\mathcal{V}_{\min}$ | 0.1 |

The first hyperparameter is the maximum depth, $\mathcal{D}_{\max}$​, which determines how deeply potential correction terms can be inserted within the expression tree. This parameter offers considerable flexibility for human-machine interaction, which is important since, in practice, deciding on a model’s complexity for a new system often relies on human judgment. In symbolic regression, it is common to impose a limit on overall complexity, $\mathcal{C}_{\max}$*,* defined as the total number of input features and mathematical operations. The maximum depth hyperparameter, should increase with the source model’s complexity. However, if $\mathcal{D}_{\max}$ is set too high, it may complicate the identification of useful correction terms. For very large expressions, one effective strategy is to start with a lower $\mathcal{D}_{\max}$ to identify which general terms need correction, and then selectively increase $\mathcal{D}_{\max}$ for those terms to achieve more precise adjustments.

The next set of hyperparameters – $\lambda_{1}$ and $\lambda_{2}$ – serve to weight the different components of the penalty term $P\left( \boldsymbol{y,\omega} \right)$ as defined in Eq. 2e in the main text of this article. The first component penalises the number of input features used in the corrections to discourage the ANN from learning corrections that will have complex symbolic representations constructed from many input features. The second component works to deactivate as many correction terms as possible by pushing their average values toward either zero or one, depending on whether the correction is applied additively or multiplicatively. These weighting factors were tuned to ensure robust performance on the set of equations listed in Table S2, which includes both the ground-truth, $g\left( \boldsymbol{y} \right)$, equations to be recovered and the expressions to be corrected, $f\left( \boldsymbol{y} \right)$. The domain for the input features in each test problem was: $-1\leq y_{i}\leq1$, where $y_{i}$ for $i\in\left\{ 1, 2, 3,4 \right\}$ denote each input feature. During the calibration process, the incorrect form $f\left( \boldsymbol{y} \right)$ served as the starting point for recovering the correct ground-truth $g\left( \boldsymbol{y} \right)$. Since all features were normalised in this study, the hyperparameter settings obtained from the experiments in Table S2 can be applied directly to other cases without necessarily needing further modification. It is also worth noting that the calibration expressions are not exhaustive. They also only contained the operators: addition, multiplication, subtraction, division and powers; exponentials and trigonometric functions were not included since these are less common in formulation and (bio)chemical reaction kinetic equations. Where exponentials are involved such as the Arrhenius or Beer-Lambert law, then it is usually better practice to provide these as fixed substructures (i.e., as functions of temperature or transmission path length, respectively).

Table S2: Set of ground-truth and initial expressions to be corrected used for calibration of the framework’s hyperparameters. A range of complexities, $\mathcal{C}$, were explored.

| $\mathcal{C}$ | Ground-Truth $g\left( \boldsymbol{y} \right)$ | Expression-to-be-Corrected $f\left( \boldsymbol{y} \right)$ |
| --- | --- | --- |
| 3 | $z=y_{1}+y_{2}$ | $z=y_{1}$ |
| 5 | $z=y_{1}+y_{2}y_{3}$ | $z=y_{1}+y_{2}$ |
| 5 | $z=y_{1}+\frac{y_{2}}{y_{3}}$ | $z=y_{1}+\frac{y_{2}}{y_{4}}$ |
| 5 | $z=y_{1}+y_{2}^{2}$ | $z=y_{1}+y_{2}$ |
| 7 | $z=y_{1}y_{2}+y_{2}y_{3}$ | $z=y_{1}+y_{2}$ |
| 7 | $z=\frac{y_{1}}{y_{2}}+\frac{y_{2}}{y_{4}}$ | $z=y_{1}+y_{2}$ |
| 7 | $z=y_{1}y_{2}+\frac{y_{2}}{y_{4}}$ | $z=y_{1}y_{2}+\frac{1}{y_{3}}$ |
| 7 | $z=y_{1}y_{2}y_{3}+y_{3}$ | $z=y_{1}y_{2}+y_{4}$ |
| 7 | $z=y_{1}y_{2}+y_{2}y_{3}$ | $z=y_{1}y_{2}+y_{2}y_{4}$ |
| 9 | $z=y_{1}y_{2}+\frac{y_{3}}{y_{1}y_{2}}$ | $z=y_{1}+\frac{y_{3}}{y_{1}}$ |
| 9 | $z=y_{1}+\frac{y_{2}+y_{3}}{y_{1}+\theta_{1}}$ | $z=y_{1}+\frac{y_{2}y_{3}+y_{3}}{y_{4}+\theta_{1}}$ |
| 9 | $z=\frac{y_{1}+y_{2}y_{3}}{y_{1}+\theta_{1}}$ | $z=\frac{y_{1}+y_{2}}{y_{1}+y_{4}}$ |
| 9 | $z=\left( y_{1}+y_{2} \right)\left( y_{1}y_{2}+y_{3} \right)$ | $z=y_{1}\left( y_{1}+y_{3} \right)$ |
| 9 | $z=\left( \frac{y_{1}}{y_{2}}+y_{3} \right)\left( y_{1}+y_{2} \right)$ | $z=\left( \frac{y_{1}}{y_{2}y_{3}} \right)\left( y_{1}+y_{2} \right)$ |
| 9 | $z=\frac{\left( y_{1}+y_{2} \right)\left( y_{1}+y_{3} \right)}{y_{4}}$ | $z=y_{1}\left( y_{1}+y_{2} \right)$ |
| 9 | $z=\frac{y_{1}+y_{2}}{y_{4}\left( y_{1}+y_{3} \right)}$ | $z=\frac{1}{y_{4}\left( y_{1}+y_{3} \right)}$ |
| 11 | $z=y_{1}y_{2}+y_{2}y_{3}+y_{3}y_{4}$ | $z=y_{1}y_{2}^{2}+y_{3}$ |
| 11 | $z=y_{1}y_{2}+\frac{y_{2}y_{3}}{y_{1}y_{4}}$ | $z=y_{1}+\frac{y_{2}y_{4}}{y_{1}y_{3}}$ |
| 11 | $z=y_{1}y_{2}+\frac{y_{2}y_{3}}{y_{3}+\theta_{1}}$ | $z=y_{1}+\frac{y_{2}y_{4}}{y_{3}}$ |
| 11 | $z=\frac{y_{1}y_{2}^{3}}{y_{3}y_{4}+\theta_{1}}$ | $z=\frac{y_{1}y_{2}^{2}}{y_{1}}$ |
| 11 | $z=\left( y_{1}y_{2}+y_{3} \right)^{2}\left( y_{3}+y_{4} \right)$ | $z=\left( y_{1}y_{2}+y_{3} \right)\left( y_{1}+y_{4} \right)$ |
| 11 | $z=\left( y_{1}y_{2}+y_{3} \right)\left( \frac{y_{1}}{y_{2}}+y_{2} \right)$ | $z=\left( y_{1}y_{2}+\frac{y_{3}}{y_{4}} \right)\left( y_{1}+y_{2} \right)$ |
| 11 | $z=y_{1}y_{2}\left( y_{3}+\left( \frac{y_{1}}{y_{2}} \right)^{2} \right)$ | $z=y_{1}y_{2}\left( \theta_{1}+\frac{y_{1}}{y_{2}} \right)$ |
| 11 | $z=\frac{y_{1}y_{2}+y_{3}y_{4}}{y_{1}y_{2}}$ | $z=\frac{y_{1}y_{2}}{y_{1}y_{2}+y_{3}y_{4}}$ |
| 11 | $z=\frac{y_{1}y_{2}}{y_{1}y_{2}+y_{3}y_{4}}$ | $z=\frac{y_{1}y_{2}+y_{3}y_{4}}{y_{1}y_{2}}$ |
| 13 | $z=y_{1}y_{2}+\left( y_{2}y_{3}+y_{3}y_{4} \right)^{2}$ | $z=y_{1}y_{3}+\left( y_{2}y_{3}+y_{3}y_{4} \right)^{3}$ |
| 13 | $z=y_{1}y_{2}+\frac{y_{2}y_{3}}{y_{1}y_{3}+y_{2}}$ | $z=y_{1}y_{4}+\frac{y_{2}y_{3}}{y_{1}+y_{2}}$ |
| 13 | $z=\frac{y_{1}y_{2}+y_{2}y_{3}}{y_{2}y_{3}+y_{4}}$ | $z=\frac{y_{1}y_{2}}{y_{2}y_{3}+\theta_{1}}$ |
| 13 | $z=\left( y_{1}y_{2}+y_{2}y_{3} \right)\left( y_{2}y_{3}+y_{4} \right)$ | $z=y_{1}y_{2}\left( y_{2}y_{3}+y_{4} \right)^{2}$ |
| 13 | $z=\frac{y_{3}\left( y_{1}y_{2}+y_{2} \right)}{y_{3}y_{4}+\theta_{1}}$ | $z=\frac{y_{1}y_{2}y_{3}+\theta_{1}}{y_{3}y_{4}}$ |
| 13 | $z=y_{1}y_{2}\left( y_{3}y_{4}+\frac{y_{1}}{y_{2}y_{4}} \right)$ | $z=y_{3}y_{4}\left( y_{1}y_{2}+\frac{y_{1}}{y_{2}y_{4}} \right)$ |
| 13 | $z=\left( y_{1}+y_{2}+y_{3} \right)\left( y_{1}y_{2}+\frac{y_{3}}{y_{4}} \right)$ | $z=y_{1}+y_{2}+y_{3}$ |
| 13 | $z=\frac{y_{1}+y_{2}+y_{3}}{y_{1}y_{2}+\frac{y_{3}}{y_{4}}}$ | $z=\frac{y_{1}+y_{2}}{y_{1}y_{2}+\frac{y_{4}}{y_{3}}}$ |
| 13 | $z=\frac{y_{1}+y_{2}}{y_{1}\left( y_{2}+y_{3}y_{4} \right)^{2}}$ | $z=\frac{\left( y_{1}+y_{2} \right)^{2}}{y_{1}\left( y_{2}+y_{3}y_{4} \right)}$ |
| 13 | $z=\left( y_{2}+y_{3}y_{4} \right)^{2}\left( y_{1}+y_{2} \right)^{3}$ | $z=\left( y_{2}+\frac{y_{3}}{y_{4}} \right)^{2}\left( y_{1}+y_{2}y_{4} \right)^{3}$ |
| 13 | $z=\left( y_{2}+y_{3} \right)^{2}\left( y_{1}+y_{2} \right)^{3}+y_{4}$ | $z=\left( y_{2}+y_{1}y_{3} \right)\left( y_{1}+y_{2} \right)^{3}$ |

The hyperparameter, $\mathcal{V}_{\min}$​, controls the number of correction terms retained after successively eliminating those deemed least significant. Specifically, $\mathcal{V}_{\min}$​, sets the minimum variance threshold for the corrections; any correction whose variance falls below this value is also removed. This precaution is designed for cases where the algorithm narrows the potential corrections down to $\mathcal{U}_{\max}$​ candidates, yet one has such a small variance that it should not be retained. Like the calibration of $\lambda_{1}$ and $\lambda_{2}$ the balance involved in selecting $\mathcal{V}_{\min}$​ was achieved by maximising the overall accuracy of the framework on the calibration expressions provided in Table S2. These hyperparameters were kept constant when the framework was subsequently applied to the bioprocess case study discussed in this research.

**S7. Question of Whether to Carry-Over Expressions During MbDoE**

Each MbDoE iteration, the source model was reset. In principle, the top-scoring transfer model from the previous MbDoE iteration could be used as a starting point for further modification. This would be valuable when modelling complex systems and the experimental design space is being expanded to include new operating variables. For example, the first few experiments could be operated at constant pH or temperature, or under the influence of one substrate, then the next few also vary pH or temperature, or introduce a second substrate with synergetic or inhibitory properties. Here, however, it was better to reset the source model with each MbDoE iteration to avoid unnecessary bias from being carried forward.
